# Is there a fair distribution of the structure of dental services in the capitals of the Brazilian Federative Units?

**DOI:** 10.1186/s12939-018-0899-5

**Published:** 2019-01-08

**Authors:** Rejane Christine de Sousa Queiroz, Ana Graziela Araújo Ribeiro, Aline Sampieri Tonello, Ana Carolina Mendes Pinheiro, José Aquino Júnior, Thiago Augusto Hernandes Rocha, Núbia Cristina da Silva, Elisa Miranda Costa, João Ricardo Nickenig Vissoci, Catherine Staton, Luiz Augusto Facchini, Erika Bárbara Abreu Fonseca Thomaz

**Affiliations:** 1 Department of Public Health, Postgraduate Program in Public Health, Federal University of Maranhão, São Luís, Maranhão Brazil; 2 Postgraduate Program in Dentistry, Federal University of Maranhão, São Luís, Maranhão Brazil; 3 Department of Public Health, Federal University of Maranhão, São Luís, Maranhão Brazil; 4 Postgraduate Program in Public Health, Federal University of Maranhão, São Luís, Maranhão Brazil; 5 Department of Public Health, Postgraduate Program in Environmental Health, Federal University of Maranhão, São Luís, Maranhão Brazil; 6 Pan American Health Organization, Brasilia Federal District Brazil, Brasília, Brazil; 7 Center for Graduate Studies and Research in Administration (CEPEAD), Faculty of Economic Sciences (FACE), Federal University of Minas Gerais, Belo Horizonte, Minas Gerais Brazil; 8 Graduate Program in Public Health, Federal University of Maranhão, São Luís, Maranhão Brazil; 9 Duke Emergency Medicine, Duke University Medical Center, Durham, NC USA; 10 Duke Global Health Institute, Duke University, Durham, NC USA; 11 Department of Social Medicine, Postgraduate Programs in Epidemiology and Nursing, Federal University of Pelotas, Pelotas, Rio Grande do Sul Brazil; 12 Department of Public Health, Postgraduate Program in Public Health, Postgraduate Program in Dentistry, Federal University of Maranhão, São Luís, Maranhão Brazil

**Keywords:** Dental care, Health services coverage, Human resources in dentistry, Equity

## Abstract

**Background:**

Brazilian Primary Care Facilities (PCF) provide primary care and must offer dental services for diagnosis, prevention, and treatment of diseases. According to a logic of promoting equity, PCF should be better structured in less developed places and with higher need for oral health services.

**Objective:**

To analyze the structure of dental caries services in the capitals of the Brazilian Federative Units and identify whether socioeconomic factors and caries (need) are predictors of the oral health services structure.

**Methods:**

This is an ecological study with variables retrieved from different secondary databases, clustered for the level of the federative capitals. Descriptive thematic maps were prepared, and structural equations were analyzed to identify oral health service structure’s predictors (Alpha = 5%). Four models with different outcomes related to dental caries treatment were tested: 1) % of PCF with a fully equipped office; 2) % of PCF with sufficient instruments, and 3) % of PCF with sufficient supplies; 4) % of PCF with total structure.

**Results:**

21.6% of the PCF of the Brazilian capitals had a fully equipped office; 46.9% had sufficient instruments, and 30.0% had sufficient supplies for caries prevention and treatment. The four models evidenced proper fit indexes. A correlation between socioeconomic factors and the structure of oral health services was only noted in model 3. The worse the socioeconomic conditions, the lower the availability of dental supplies (standard factor loading: 0.92, *P* = 0.012). Estimates of total, direct and indirect effects showed that dental caries experience observed in the Brazilian population by SB-Brasil in 2010 did not affect the outcomes investigated.

**Conclusion:**

Material resources are not equitably distributed according to the socioeconomic conditions and oral health needs of the population of the Brazilian capitals, thus contributing to persistent oral health inequities in the country.

## Introduction

In Brazil, the access to oral health services happens through the public or private services providers; the latter directly paid by the users or by health insurance companies [1]. Public dental services are mainly provided through oral health primary care teams (OHT). OHT are publicly funded healthcare providers offering care free of charge to prevent diseases and promote oral health [2].

The insertion of OHTs in the Family Health Strategy established a new paradigm of Primary Health Care (PHC) planning and programming. Taking into consideration the oral health populational needs was one of the essential public care initiatives in the reorganization of health actions from the Brazilian Public Health System (SUS, acronym in Portuguese) [1–8].

The number of OHT has been increasing over the years in Brazil [7, 9, 10]. Despite the increased oral health actions and higher use of dental services, both organizational and geographical access barriers are still a significant challenge [5, 6, 11–13]. Aspects related to users’ perceived need about services provided, as well as the number, geographical location, and type of health equipment are important factors that highlight the inequality between supply and demand [13], thus revealing persistent social inequities in oral health.

Studies have indicated that variables, such as low-income levels [14], vulnerable age groups (children and elderly), greater disease severity [15, 16], residing in rural areas [16] and poor financial investments in health [7] may be associated with lower population access to dental services [14]. The distribution of oral health resources is often inversely proportional to the needs of the population [17]. However, their scope is not national. They consider incipient aspects of the structure of oral health services, and none of them used structural equation modeling techniques to test direct and indirect associations between variables of interest.

Efforts are also required to reduce access inequalities, improve the care process and use epidemiological results in oral health for PHC planning [18]. One of the first steps in this direction is to expand and improve the structure of the services offered, increasing access and providing structural conditions for the reorganization of the care model. However, while data available on the structure of PHC is available in Brazil since 2002 [19], no studies have yet analyzed whether the structure of oral health services is oriented by the principle of equity, that is, whether service delivery is better in less developed areas and more demand-driven locations. Thus, this study aims to analyze the structure of oral health services in the capitals of the Brazilian Federal Units, identifying socioeconomic and oral health demand predictors.Our hypothesizes is that there is a relationship of inequity between the services provided and population’s demand.

## Material and methodology

### Study design and location

The present work can be categorized as an ecological study, with a descriptive and analytical approach, using different secondary sources of data. The unit of analysis consisted of the 27 capitals of the Brazilian States.

### Theoretical model

**Fig. 1:**
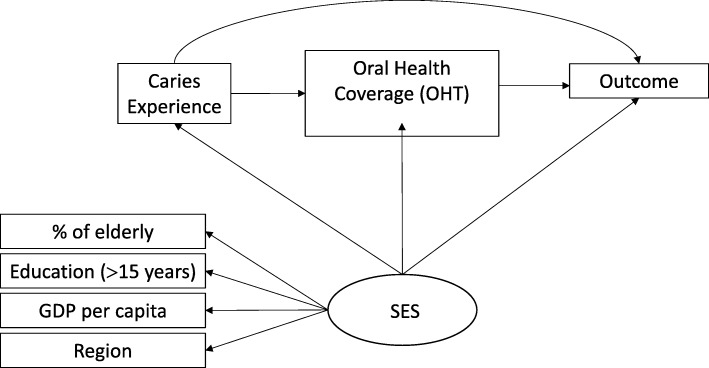
Theoretical model of predictors of the structure of oral health services in the capitals of the Brazilian Federative Units. Outcome: Structure of oral health services

A hypothetical model was created (Fig. 1) to analyze whether the socioeconomic status (latent variable “SES”) and the need for oral health services (variables observed “Caries experience”) have a direct or indirect impact on oral health services structure (outcome). Three indirect pathways were tested for the association between socioeconomic conditions and oral health services structure: 1) SES → Caries → Outcome; 2) SES → Caries → OHT coverage → Outcome, and 3) SES → OHT coverage → Outcome. A single indirect causal pathway for the association between caries experience and the structure of oral health services was tested: 1) Caries → OHT coverage → Outcome.

There are indications that worse socioeconomic conditions are associated with greater caries experience in the population [20], and greater OHT coverage [21, 22]. However, there is a lack of evidence of a possible relationship with aspects related to physical structure [17, 21] of oral health services (outcome).

Bordin and Fadel [23] argue that the experience of caries in the population should influence the coverage of OHT in municipalities. In turn, Corrêa and Celeste [7] report that higher coverage of OHT should imply better indicators of oral health services’ structure. Therefore, OHT coverage in Brazil is considered to be a determinant of service structure indicators [24]. The main reason for this is due to OHT’s accreditation and implementation process that is subsidized by the federal public power through financial transfers (BRASIL, 2000) and the supply of dental equipment [25].

The latent variable “SES” was composed by four different variables: population proportion of elderly people; proportion of the population with more than 15 years of study; Gross Domestic Product (GDP) per capita and region.

The region is a geopolitical construct that aggregates municipalities with similar geographical conditions, cultural perspective, economic activities and other characteristics that help define their socioeconomic status. Considering the region role we considered this domain as a relevant variable to compose the latent variable SES. This consideration is essential once the implementation of PHC is heterogeneous in the different geographic areas. We can observe differences considering the local context and present singularities that result in the diversity of structures and service provision [24].

A possible direct impact of caries’ experience on the population vis-à-vis the surveyed outcomes is also advocated by some authors, insofar as these data should support the implantation and implementation of the services [26].

### Study variables/indicators and data sources

The socioeconomic variables were: a) Percentage of people aged 60 and over, in the total population [27]; b) Proportion of the population with more than 15 years of study, calculated as the number of people who studied 15 years and over diveded by the total population multiplied by 100 [28]; c) Gross domestic product (GDP) per capita, calculated as the average aggregate value per person of the final goods and services produced in a given geographic space [27]; and d) Geopolitical region [27]. All these variables were considered in the year of 2010.

Data from the caries experience of the Brazilian population derived from the results of the SB Brasil 2010 survey [28]. The caries indicator was built considering the number of people with experience of dental caries at the time of examination divided by the total number of people evaluated multiplied by 100. The resulting indicator reflects the proportion of people with caries experience in each capital. Data on OHT coverage were obtained from the Primary Care Department of the Ministry of Health (DAB/SAS/MS) at the year of 2011.

Four different outcomes regarding oral health services structure were considered: 1) Proportion of Primary Care Facilities (PCF) with a fully equipped dental office (Statistical model 1); 2) Proportion of PCF with a sufficient number of all instruments (Statistical model 2); 3) Proportion of PCF with a sufficient stock of all necessary dental supplies (Statistical model 3); and 4) A latent variable composed by all the previous outcomes (Statistical model 4). These data were obtained from the first PCF census carried out along with the first cycle of the External Evaluation of the Program of Improvement of Access and Quality of Primary Care (PMAQ-AB) in 2012 [3, 29].

PCF was defined as having a fully equipped dental office when it had all the eight following items – a room for OHT, dental chair, compressor, delivery systems, stool, reflector, auxiliary unit or assistant’s vacuum group and photopolymerizer (outcome of model 1). For model 2, the outcome was the proportion of PCF that had all the 8 following restorative dental instruments: Dycal applicator, dentin brushers, resin insertion trowel, tweezers, mirror, explorer, glass plate and carpule syringe. For model 3, the outcome was the proportion of PCF that has all the 12 following dental restorative supplies: Acid and tapes, cotton, local anesthetics, various drills, restorative cement, PPE, fluoride, gauze, temporary restorative material, carbon paper, sealants, resins and box of piercing material.

### Data review

Absolute frequencies, as well as means (± standard deviations), were estimated for the study variables. For analysis, due to the wide variance, the variables proportion of the elderly population, PHC financing and GDP per capita were categorized into quartiles. The other variables were included in the model in its original form.

Georeferenced maps were developed for the sociodemographic conditions of capitals through the ArcGIS® program, version 10.2. The georeferencing of population health situation data is pointed out as an essential tool in the process of evaluation of PHC actions. It enables the development of adequate diagnoses of the reality in which health teams are inserted and enhances the identification and understanding of positive and problematic realms of the work process, as well as possible access barriers [30].

Structural equations were analyzed using MPlus® software, version 7.4 (Muthen & Muthen, North Carolina, United States), considering four different models, one for each outcome and one final model including the three outcomes at the same time (that we called ‘total structure’). The structural equations model consists of two sub-models: the measurement model, which establishes how the constructs are measured, and the structural model, which analyzes the theoretical model as a whole, where associations between the variables are determined. The latent variables that define constructs are shown through circles or ellipses, and the observed variables are represented by squares or rectangles. The latent variable is elaborated in the measurement model, where indicators of this variable are specified. A good latent variable has convergent validity, showing that its indicators measure the same construct, verified by high-value factor loadings (higher than 0.50). Also, there should be discriminant validity, that is, correlations between indicators should not be excessively high (> 0.95), demonstrating that each indicator measures different aspects of the construct [31].

Standard factor loadings (SFL) were interpreted according to parameters used by Kline [32] and Cook, Kallen, and Amtmann [33﻿], where values close to 0.10 indicate a small impact, 0.30 an average impact and values greater than 0.50 indicate a strong impact. The models tested were evaluated by fit indexes, including RMSEA (Root Mean Square Error of Approximation), CFI (Comparative Fit Index), TLI (Tucker-Lewis Index) and WRMR (Weighted Root Mean Square Residual). Acceptable values were RMSEA < 0.06; CFI/TLI > 0.95; WRMR < 1.0 [36]. The chi-square test was used to determine the value of P (X^2^), and the lowest values were used as a reference.

## Results

**Fig. 2:**
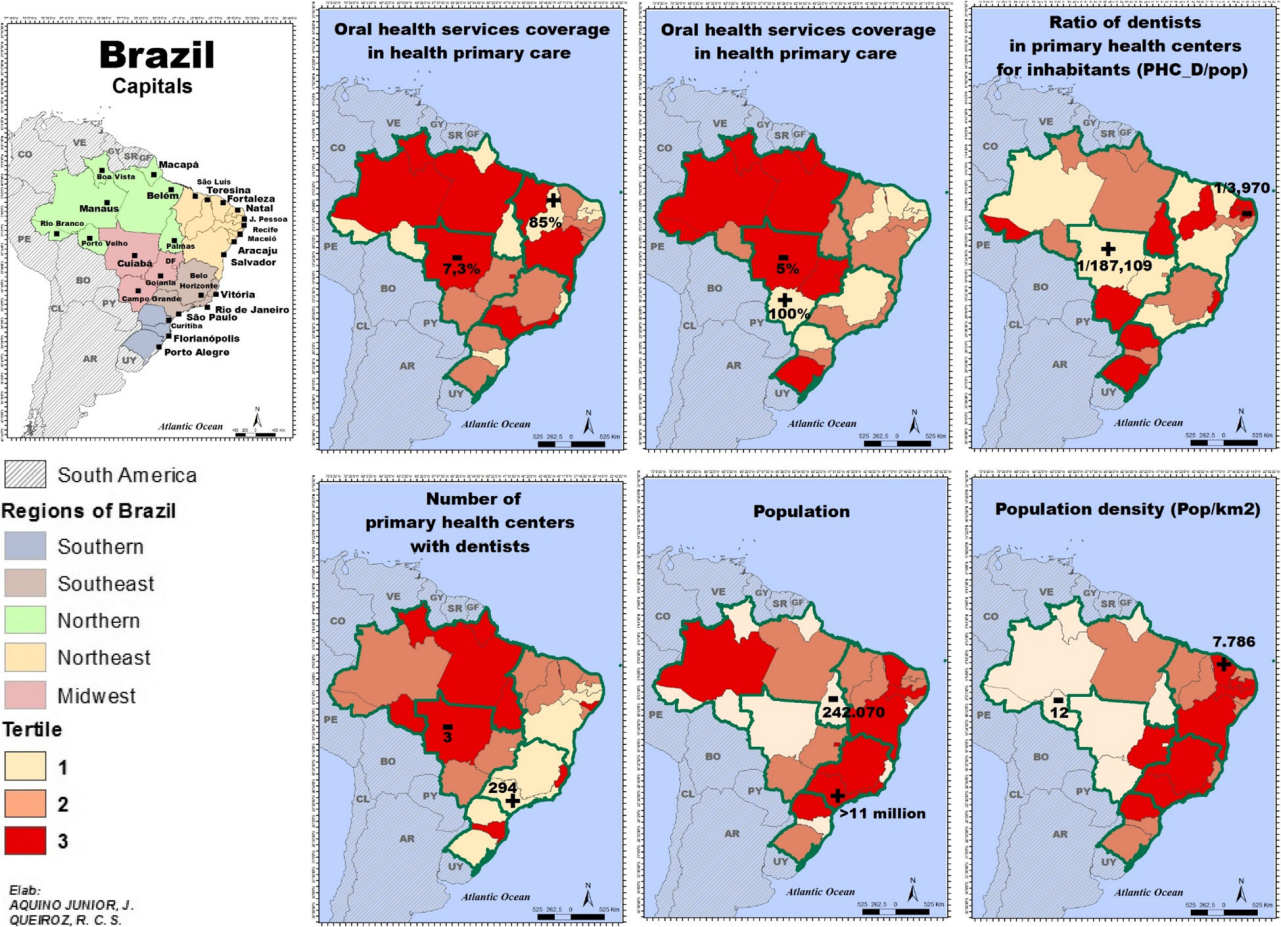
Socioeconomic characterization and distribution of oral health services in Brazilian capitals. 2010

The study sample consisted of the 27 capitals of the Brazilian Federative Units. Figure 2 shows the sociodemographic characterization, as well as the distribution of oral health services by capital. São Paulo was the most populous capital (11 million inhabitants) and the capital with the lowest population was Palmas (242,070 inhabitants). The highest population density was recorded in Fortaleza (7786 inhabitants per km^2^) with Porto Velho with the lowest density comprising 12 inhabitants per km^2^.

Capital cities with the lowest and highest number of PCF per 100 thousand inhabitants were Rio de Janeiro (2.8) and João Pessoa (20.3), respectively. Porto Alegre (15%) and Rio de Janeiro (14.9%) were the capitals with the highest proportion of elderly, while Palmas had the lowest (4.4%). Porto Alegre (80.1%) and Belo Horizonte (80.1%) presented the highest proportion of people with greater than 15 years of study, while Macapá (65.6%) showed the lowest. The GDP per capita varied from R$ 15,889.20 in Maceió to R$ 72,975.10 Vitória, followed by Brasília (R$ 61,876.10) and São Paulo (R$ 46,883.00). Florianópolis had the smallest part of the population that has or had experience of caries (73.3% of the population – that means 26.7% of the surveyed population of this capital were caries free), while Porto Velho was the capital with the highest caries population (93.5%). Rio Branco, Belém, São Luís, Natal, Maceió, Curitiba, and Cuiabá did not show a PCF with a fully equipped dental office, and Belo Horizonte was the capital with the highest proportion of fully equipped PCF dental offices (74.7%). Two capitals, namely, Boa Vista and Belém, did not have enough dental instruments to perform restorative dentistry procedures, while Cuiabá was the only capital that had 100% of its health facilities with complete dental instruments. Boa Vista, Macapá, São Luís, Teresina, and Maceió did not show facilities with complete restorative dental supplies, while Cuiabá once again had 100% of its facilities with all the necessary supplies (Table 1).

**Table 1:** Description of study variables. Brazilian capitals, 2010–2012

	N° PCF/100,000 inhabitants	% elderly	% education > than 15 years	GDP per capita (R$)	% population with caries	OHT Coverage (%)	% Dental office^a^	% Dental instruments^b^	% Dental supplies^c^
Porto Velho-RO	9.9	5.6	70.1	28,842.40	93.5	64.9	5.9	52.9	64.7
Rio Branco-AC	19.2	6.4	67.7	17,958.00	89.9	51.3	0.0	52.4	23.8
Manaus-AM	12.1	6.0	69.3	29,803.80	89.4	27.8	2.1	77.9	15.7
Boa Vista-RR	12.1	5.2	66.5	19,653.60	90.6	27.6	27.3	0.0	0.0
Belém-PA	5.2	9.3	75.7	17,451.80	89.3	16.9	0.0	0.0	4.2
Macapá-AP	11.8	5.2	65.6	17,863.00	88.7	55.6	4.2	8.3	0.0
Palmas-TO	13.6	4.4	68.7	20,085.40	89.3	59.4	41.9	87.1	77.4
São Luís-MA	4.8	7.7	74.3	21,827.90	87.0	23.1	0.0	20.6	0.0
Teresina-PI	9.9	8.5	74.9	16,071.20	83.6	85.0	44.4	33.3	0.0
Fortaleza-CE	3.6	9.7	75.7	18,259.40	87.5	45.3	66.7	10.0	16.7
Natal-RN	6.5	10.4	76.7	21,608.10	86.0	50.8	0.0	23.5	8.8
João Pessoa-PB	20.3	10.3	75.2	18,645.40	91.5	83.9	3.1	35.7	16.3
Recife-PE	8.9	11.8	77.8	27,272.80	82.9	30.8	9.1	70.2	57.0
Maceió-AL	3.9	8.5	73.0	15,889.20	87.2	31.2	0.0	13.3	0.0
Aracaju-SE	7.3	9.1	75.2	21,310.00	74.3	43.9	7.4	22.2	7.4
Salvador-BA	3.9	9.3	77.9	17,436.40	76.6	14.8	48.4	38.7	6.5
Belo Horizonte-BH	6.1	12.6	80.1	31,004.10	78.8	47.7	74.7	45.9	56.8
Vitória-ES	7.8	12.0	79.2	72,975.10	77.5	56.7	36.4	95.5	86.4
Rio de Janeiro-RJ	2.8	14.9	79.2	39,405.30	79.8	25.0	14.5	85.5	68.0
São Paulo-SP	3.8	11.9	78.0	46,883.00	83.5	17.3	57.1	41.3	10.8
Curitiba-PR	5.7	11.3	78.5	39,243.00	84.4	44.4	0.0	87.9	73.7
Florianópolis-SC	11,3	11.5	79.2	31,998.90	73.3	62.0	45.5	93.2	56.8
Porto Alegre-RS	14.5	15.0	80.1	38,056.80	81.3	33.6	26.6	66.4	25.8
Campo Grande-MS	7.2	9.9	74.8	23,796.90	86.9	42.4	2.9	61.8	11.8
Cuiabá-MT	11.4	8.1	75.1	28,837.70	85.8	7.3	0.0	100.0	100.0
Goiânia-GO	6.3	9.6	77.1	28,343.10	80.6	35.6	20.3	25.4	10.2
Brasília-DF	5.5	7.7	73.6	61,876.10	76.9	23.5	44.6	16.1	10.7
BRAZIL	6.0	9.3	74.8	28,607.30	84.3	41.0	21.6	46.9	30.0

^a^Proportion of Primary Health Care Facilities in the municipality who have all the eight types of equipment in the dental office (a room for OHT, dental chair, compressor, delivery systems, stool, reflector, auxiliary unit or assistant’s vacuum group and photopolymerizer)

^b^Proportion of Primary Health Care Facilities in the municipality who have all the eight types if instrument (dycal applicator, dentin brushers, resin insertion trowel, tweezers, mirror, explorer, glass plate and carpule syringe)

^c^Proportion of Primary Health Care Facilities in the municipality who have all the 12 dental restorative supplies (acid and tapes, cotton, local anesthetics, various drills, restorative cement, PPE, fluoride, gauze, temporary restorative material, carbon paper, sealants, resins and box of piercing material)

The capital with the highest coverage of oral health services was Teresina (85%), in the Northeast, while Cuiabá (7.3%), in the Midwest, showed the lowest coverage rate. The proportion of PCF with dentists ranged from 5% in Cuiabá to 100% in Campo Grande. The best proportion of dentists per inhabitant was found in the Northeast region, in the city of João Pessoa (1 dentist per 3970 inhabitants), while the lowest was in Cuiabá (1 dentist per 187,109 inhabitants) (Fig. 2).

The four models investigated had well-fitted indexes. While comparing the ideal hypothetical model with the proposed models, the chi-square test did not show significant differences (*P* > 0.05), indicating model adequacy. RMSEA’s values were lower or near the acceptable maximum (0.06), showing a similarity among the models examined and the hypothetical model. In all three models, CFI and TLI indexes are close to or greater than 0.95, and WRMR is less than 0.95 (Table 2).

**Table 2:** Model adjustment measures. Brazilian capitals, 2010–2012

Fit Indexes	Model 1 (Outcome: Fully equipped office)	Model 2 (Outcome: Sufficient instruments)	Model 3 (Outcome: Sufficient supplies)	Model 4 (Outcome: All previous outcomes)
	Values	Values	Values	Values
N° of free parameters	29	26	26	33
Degrees of freedom	17	11	11	23
X^2a^	13.76	9.22	10.64	24.32
*P*-value of X^2^	0.684	0.322	0.474	0.387
RMSEA^b^	< 0.001	< 0.001	< 0.001	0.040
90% CI^c^ of RMSEA	0.000–0.139	0.000–0.175	0.000–0.197	0.000–0.167
CFI^d^	0.999	0.999	0.998	0.987
TLI^e^	0.999	0.999	0.997	0.982
WRMR^f^	0.273	0.237	0.264	0.453

^a^Chi-Square Test (reference: lowest value)

^b^Root Mean Square Error of Approximation (reference: below 0.05)

^c^Confidence Interval at 90% (reference: below 0.08)

^d^Comparative Fit Index (reference: below 0.9)

^d^Tucker Lewis Index (reference: above 0.9)

^f^Weighted Root Mean Square Residual (reference: above 1.0)

Most of the variance of the SES construct was explained by the years of study, by the proportion of the elderly population and by the geopolitical region. The per capita GDP of the capitals presented the lowest coefficients of determination in the four models, but still with values above 0.3 (Table 3).

**Table 3:** Residual variances and coefficients of determination. Brazilian capitals, 2010–2012

Factor/ Variables	Model 1^a^		Model 2^b^		Model 3^c^		Model 4^d^	
	R^2^	Residual variance	R^2^	Residual variance	R^2^	Residual variance	R^2^	Residual variance
SES								
% elderly	0.677	0.323	0.651	0.349	0.622	0.378	0.628	0.372
% education greater than 15 years	0.894	0.106	0.844	0.156	0.810	0.190	0.791	0.209
GDP per capita	0.349	0.651	0.420	0.580	0.434	0.566	0.512	0.488
Geopolitical region	0.592		0.610		0.645		0.620	

*SES* Socioeconomic status, *R*^*2*^ Coefficient of determination

^a^Outcome: % PCF w/ fully equipped office

^b^Outcome: % PCF w/ sufficient instruments

^c^Outcome: % PCF w/ sufficient supplies

^d^Outcome: A latent variable constructed from the three previous outcomes

**Fig. 3:**
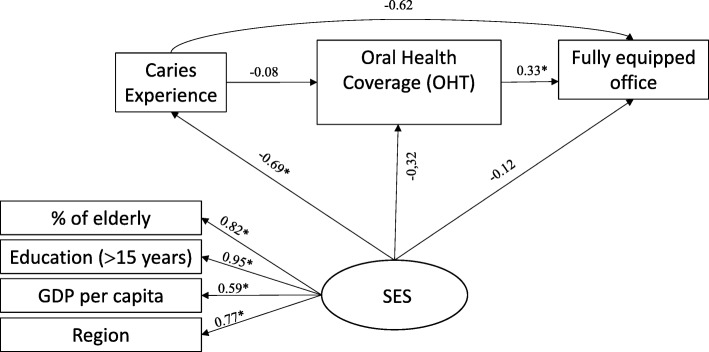
Standard factor loadings in a theoretical model explaining the proportion of fully equipped offices (Model 1) in Brazilian capitals, 2010–2012

For model 1, all SFLs that formed the SES construct (socioeconomic status) were higher than 0.50 and statistically significant (*P* < 0.05), indicating good convergent validity, and were also lower than 0.95, indicating good discriminant validity. There was a negative correlation between SES and caries experience (SFL = − 0.69, *P* < 0.001). However, dental caries experience in 2010 was not correlated with ESB coverage (SFL = − 0.08, *P* = 0.792), nor with the presence of at least eight essential items of the dental office (SFL = − 0.62, *P* = 0.076) (Fig. 3).

**Fig. 4:**
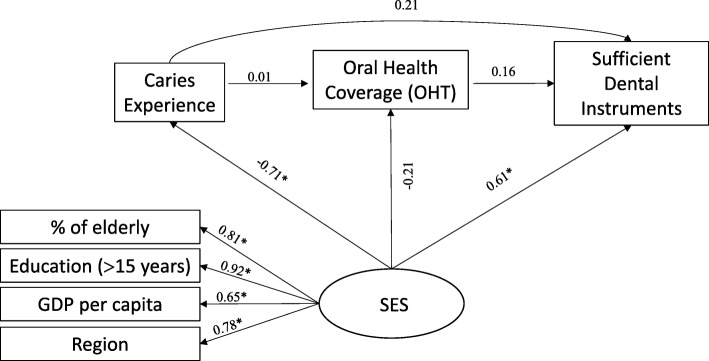
Standard factor loadings in a theoretical model explaining the proportion of PCF with sufficient dental instruments (Model 2) in the Brazilian capitals, 2010–2012

**Fig. 5:**
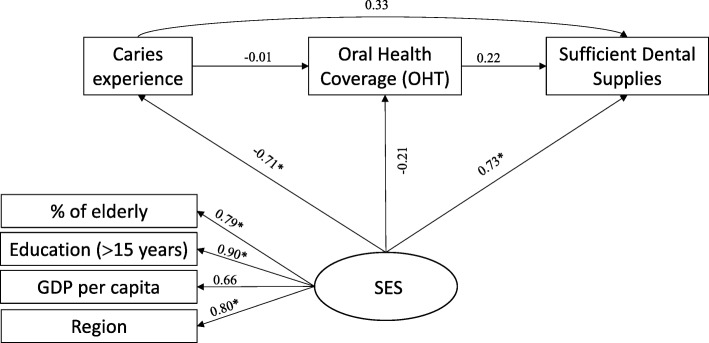
Standard factor loadings in a theoretical model explaining the proportion of PCF with sufficient dental supplies (Model 3) in the Brazilian capitals, 2010–2012

**Fig. 6:**
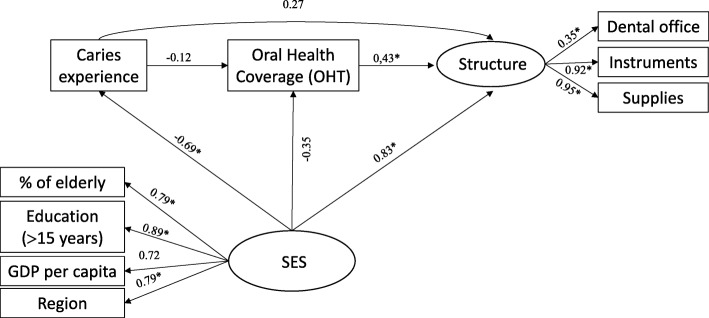
Standard factor loadings in a theoretical model explaining the proportion of fully equipped offices (Model 4) in Brazilian capitals, 2010–2012

In models 2, 3 and 4, SFLs also indicated good convergent and divergent validity in the construction of the latent variable (SES). A negative correlation was also observed between the SES and the caries experience in all models. Similarly to model 1, there was also no correlation between the proportion of people with dental caries in Brazilian capitals and the structure of instruments and oral health supplies necessary for the treatment of the disease, even when the three outcomes were considered together (model 4). However, capitals with the best SES evidenced a greater proportion of provision of dental instruments (SFL = 0.61, *P* = 0.010), dental supplies (SFL = 0.73, *P* = 0.008) and the structure (three outcomes together (SFL = 0.83, *P* = 0.001) (Figs. 4, 5 and 6).

Estimates of total, direct and indirect effects showed that dental caries experience verified in the Brazilian population by SB-Brasil in 2010 had no impact on the total structural outcomes investigated in 2012/13. However, SES had a significant total effect on the availability of dental instruments (SFL = 0.431; *P* = 0.010), supplies (SFL = 0.449, *P* = 0.012) and total structure (SFL = 0.532, *P* = 0.002), as well as a significant direct effect on the availability of dental instruments (SFL = 0.612, *P* = 0.010), supplies (SFL = 0.727, P = 0.008) and total structure (SFL = 0.834, P = 0.001) (Table 4).

**Table 4:** Standardized estimates of total, direct and indirect effects of the predictive model of oral health structure in PHC. Brazilian capitals, 2010–2012

	Model 1		Model 2		Model 3		Model 4	
	SFL^a^	*P*	SFL^a^	*P*	SFL^a^	*P*	SFL^a^	*P*
Caries➔Outcome								
Total effect	−0.645	0.053	0.209	0.316	0.331	0.102	0.219	0.316
Direct effect	−0.620	0.076	0.209	0.323	0.331	0.109	0.269	0.189
Indirect total effect	−0.025	0.803	< 0.01	0.996	< 0.01	0.997	−0.051	0.642
Specific indirect effect								
Caries➔Coverage➔Outcome	−0.025	0.803	< 0.01	0.996	< 0.01	0.997	−0.051	0.642
SES➔Outcome								
Total effect	0.221	0.248	0.431	0.010	0.449	0.012	0.532	< 0.002
Direct effect	−0.115	0.750	0.612	0.010	0.727	0.008	0.934	0.001
Indirect total effect	0.337	0.314	−0.181	0.280	−0.279	0.142	−0.302	0.130
Specific indirect effects								
SES➔Coverage➔Outcome	−0.107	0.386	−0.034	0.685	−0.046	0.645	−0.150	0.314
SES➔Caries➔Outcome	0.426	0.155	−0.147	0.327	−0.233	0.147	−0.187	0.199
SES➔Caries➔Coverage➔Outcome	0.017	0.804	< 0.01	0.996	< 0.01	0.997	0.035	0.646

*SFL* Standard factor loading, *SES* Socioeconomic status construct

Significant *p*-value (*p* ≤ 0.05) in bold

^a^Reference for standard factor loading: small effect < 0,10; Medium – around 0.30; Large > 0.50

## Discussion

The lowest values of OHT coverage and proportion of PCF with dental surgeons were found in the Northern region of the country. Similarly, some of the worst socioeconomic indicators are recorded in the Northern region, such as lower schooling rate and lower per capita GDP. A negative correlation was identified between the socioeconomic status of the population and dental caries in all models evaluated. The socioeconomic condition was also associated with the availability of dental supplies necessary for the prevention and treatment of dental caries, but not with OHT coverage. Capitals with a higher prevalence of the population with dental caries were not those that showed a more extensive availability of material resources for the prevention and treatment of the disease.

Access to health care can be measured by the search for and use of services. However, the access cannot be explained exclusively by the need, but also by the perception, individual values, organization, health system financing, sociodemographic characteristics, among others factors [34, 35]. Even with a health system guided by the principles of universal, equitable and impartial access, Brazil is a country marked by socioeconomic inequities with enormous consequences for health, access, and use of dental services [21–23, 36, 37].

Several studies pointed out to a reduction in caries’ rates in several age groups [14, 20, 38]. However, this downswing has not been taking place equally among Brazilian regions and socioeconomic groups [39].

In our study, one can observe the persistent highest proportions of the population with caries experience in geopolitical regions with worse socioeconomic indicators. In our analysis, the importance of geopolitical regions can also be demonstrated by the coefficient of determination of the variables underlying the socioeconomic status (SES) construct as the main responsible for SES variance in all models investigated.

The North and Northeast regions showed the highest indicators of dental caries prevalence, while the South and Southeast had the best indexes, as has been verified in other analyses [14, 23]. There was a strong negative correlation in all the analyzed models between socioeconomic conditions and caries experience in the population in 2010, showing that populations with lower socioeconomic levels are those most affected by dental caries, as reported in other works [14, 22].

High prevalence rates of dental caries presuppose the need for a service structure with OHT coverage, adequate physical facilities, sufficient supplies and dental professionals capable of meeting the demand and promoting resolution of oral health problems. However, in our study, no correlation was found amongst caries experience in the population of the 27 Brazilian capitals with coverage of oral health teams. Bordin and Fadel found that the performance of epidemiological markers of dental caries was inversely related to favorable oral health indexes in regions with higher coverage of OHT [23]. Other study conducted in the South Brazilian region identified that the ratio between exodontia of permanent decayed teeth and individual dental procedures in primary care was higher as long as the coverage of OHT [21]. This evidences concern with the disease, while cure-oriented, in the places covered by primary care.

The relationship between caries experience and adequate physical structure, with fully equipped dental office, sufficient instruments, and supplies in the PCF was not evidenced in our analysis. Only socioeconomic status affected the sufficient stock of supplies, nonetheless, in the opposite direction to equity.

Junqueira et al. [17] evaluated the influence of social indicators on the health-disease process and whether these indicators modulated municipal public oral health services in the state of São Paulo using bivariate analysis. They found that the worse the illiteracy rates, the insufficient income and the poorer the living conditions, higher the number of dental equipment registered by SUS Outpatient Information System (SIA/SUS) in the municipal public services. This result differs from the findings of our study, evidencing among municipalities differences in the management of public resources. It may be that the São Paulo results showed a singularity, exhibiting a pattern of supply provision according to the needs of the population, indicating more equity in their health system, but it is also necessary reflecting methodological differences between both investigations. Our study evaluated only capitals, while these authors considered the state. There is evidence demonstrating problems in the implementation of PHC in large capitals when compared to small municipalities [40-43]. Additionally, the authors of previous studies did not adjust the models for potential confounders, such as the need for treatment.

Fischer et al. (2010), identified that the municipalities of the Brazilian South region with the worst socioeconomic conditions (municipal HDI, Gini index, indigence and proportion of the population residing in rural areas) also had the worst conditions of dental services delivery, similar to findings of our investigation [21].

Despite the contributions highlighted our study has limitations inherent to efforts that use secondary data, such as possible data inconsistencies. There were different sources, such as IBGE, UNDP, Epidemiological Survey of Oral Health (SB Brazil), SUS PHC Information System (SIAB), as well as the first OHT census of the Health Ministry. Such wide range of databases collected through different methods may occasionally result in biases due to poor recording and quality control. However, all of them are bases of the national official information systems, which have shown quality improvements over time [44–46].

Generally, health problems are analyzed as a response variable. In this study, we investigated whether one of the most important oral health problems (dental caries) was in some way considered by the Brazilian authorities to expand the service offered in places with the highest health need. Caries was considered an explanatory variable. Considering caries as an outcome could have resulted in reverse causality issues. However, in our study, the data related to exposure (in 2010) were from a period before the outcome data (in 2012/13), reducing the problem of reverse causality due to the temporal precedence of the exposure. However, the time between the diagnosis of needs and the diagnosis of facilities seems to be too short to see consistent changes in the organization of the Health Units. Thus, new assessments of the PCF structure are necessary.

According to data from the Department of Primary Care, Ministry of Health (DAB / SAS / DAB), there were 20,424 OHT implanted in 2010 (year of the last epidemiological survey on oral health in Brazil, SB-Brasil). In 2012/13, according to information available from the same source, there were 22,203 OHT implanted, indicating an increase of approximately 2.5% in oral health coverage, which consequently involves an increase in the structure of services offered.

In turn, the use of the structural equations model in the design of our study, identifying socioeconomic and oral health need predictors, was considered a strength of this work. This analysis can highlight the social context in the influence of health – both directly and indirectly – and ascertain equity in the organization and distribution of the structure of oral health services, supporting the construction of public health policies in dentistry.

The elaboration of illustrative maps, georeferencing the distribution of variables among Brazilian capitals offers a broad view of regions and contributes to support the equity discussion. The capability to better analyze the sociodemographic characterization and distribution of oral health services in the Brazilian capitals helps to identify regions with higher needs for health investment.

## Conclusion

We conclude that the structure of dental services of Brazilian Basic Health Care Facilities was not well organized according to the principle of equity. The results of the epidemiological survey of oral health in 2010 have not yet caused sufficient changes in the organization/distribution of the structure of public oral health services in the Brazilian capitals. It is necessary to increase the availability of material resources according to the epidemiological and socioeconomic indicators, thus observing the principle of equity, one of the guiding pillars of the Unified Health System.

## Abbreviations

CFI: Comparative Fit Index
FHS: Family Health Strategy
GDP: Gross domestic product
HDI: Human Development Index
IBGE: Brazilian Institute of Geography and Statistics
PCF: Primary Care Facilities
PHC: Primary Health Care
PMAQ-AB: Program of Improvement of Access and Quality of Primary Care
PNAD: National Household Sample Survey
RMSEA: Root Mean Square Error of Approximation
SES: Socioeconomic status
SFL: Standard factor loadings
TLI: Tucker-Lewis Index
WRMR: Weighted Root Mean Square Residual
